# Clinical outcomes and risk-factor analysis of the Ponseti Method in a low-resource setting: Clubfoot care in Haiti

**DOI:** 10.1371/journal.pone.0213382

**Published:** 2019-03-14

**Authors:** Rameez A. Qudsi, Faith Selzer, Stephen C. Hill, Ariel Lerner, Jean Wildric Hippolyte, Eldine Jacques, Francel Alexis, Collin J. May, Robert B. Cady, Elena Losina

**Affiliations:** 1 Orthopaedic and Arthritis Center for Outcomes Research, Department of Orthopaedic Surgery, Brigham and Women’s Hospital, Boston, Massachusetts, United States of America; 2 Policy and Innovation Evaluation in Orthopedic Treatments Center, Department of Orthopaedic Surgery, Brigham and Women’s Hospital, Boston, Massachusetts, United States of America; 3 Department of Orthopaedic Surgery, Boston Children’s Hospital, Boston, Massachusetts, United States of America; 4 Harvard Combined Orthopaedic Residency Program, Boston, Massachusetts, United States of America; 5 Harvard Medical School, Boston, Massachusetts, United States of America; 6 Boston University School of Medicine, Boston, Massachusetts, United States of America; 7 Hôpital de l’Université d’Etat d’Haiti, Port-au-Prince, Haiti; 8 CURE Clubfoot, Port-au-Prince, Haiti; 9 Department of Orthopaedic Surgery, Adventist Hospital, Diquini, Haiti; 10 Departments of Orthopaedics and Pediatrics, Upstate Medical University, Syracuse, New York, United States of America; 11 Department of Biostatistics, Boston University School of Public Health, Boston, Massachusetts, United States of America; University of Pennsylvania Perelman School of Medicine, UNITED STATES

## Abstract

**Purpose:**

The Ponseti Method has dramatically altered the management of clubfoot, with particular implications for limited-resource settings. We sought to describe outcomes of care and risk factors for sub-optimal results using the Ponseti Method in Haiti.

**Methods:**

We conducted a records review of patients presenting from 2011–2015 to a CURE Clubfoot clinic in Port-au-Prince, Haiti. We report patient characteristics (demographics and clinical), treatment patterns (cast number/duration and tenotomy rates), and outcomes (relapse and complications). We compared treatment with benchmarks in high-income nations and used generalized linear models to identify risk factors for delayed presentation, increased number of casts, and relapse.

**Results:**

Amongst 168 children, age at presentation ranged from 0 days (birth) to 4.4 years, 62% were male, 35% were born at home, 63% had bilateral disease, and 46% had idiopathic clubfeet. Prior treatment (RR 6.33, 95% CI 3.18–12.62) was associated with a higher risk of delayed presentation. Risk factors for requiring ≥ 10 casts included having a non-idiopathic diagnosis (RR 2.28, 95% CI 1.08–4.83) and higher Pirani score (RR 2.78 per 0.5 increase, 95% CI 1.17–6.64). Female sex (RR 1.54, 95% CI 1.01–2.34) and higher Pirani score (RR 1.09 per 0.5 increase, 95% CI 1.00–1.17) were risk factors for relapse. Compared to North American benchmarks, children presented later (median 4.1 wks [IQR 1.6–18.1] vs. 1 wk), with longer casting (12.5 wks [SD 9.8] vs. 7.1 wks), and higher relapse (43% vs. 22%).

**Conclusions:**

Higher Pirani score, prior treatment, non-idiopathic diagnosis, and female sex were associated with a higher risk of sub-optimal outcomes in this low-resource setting. Compared to high-income nations, serial casting began later, with longer duration and higher relapse. Identifying patients at risk for poor outcomes in a low-resource setting can guide counseling, program development, and resource allocation.

## Introduction

Congenital clubfoot is one of the most common musculoskeletal deformities at birth, affecting 1–2 babies per 1000 live births.[1–3] This accounts for approximately 150,000 to 200,000 newly affected children annually worldwide, 80% of whom are believed to be born in low and middle-income countries (LMIC).[3–5] A recent meta-analysis of clubfoot in LMIC finds an incidence in African regions of 1.11 per 1000 and in the Americas of 1.74 per 1000, projecting 43 new babies born with clubfoot each year per million population in Africa and 30 per million in the Americas. [6] Without treatment, such children may suffer life-long deformity, disability, and profound social stigma in many cultures impeding access to education and productivity.[5, 7, 8]

The development of a successful, non-operative treatment program by Dr. Ponseti in the mid 20^th^ century provided a great impetus for universal treatment of clubfoot.[9] Many programs have emerged in the developing world demonstrating the successful implementation of the Ponseti technique [3, 10–14]; however, there remains a host of challenges related to delayed presentation, barriers to care, loss to follow-up, extended casting, non-compliance, and high relapse/recurrence rates.[4, 15–17]

While established programs are known in Asia, Africa, and South America, there have been no published reports of clubfoot programs from the Caribbean region. Haiti in particular faced unique challenges in the wake of a 7.0 magnitude earthquake in January 2010. The clubfoot program had its beginnings with initial work by Dr. Kaye Wilkins from the United States and local Haitian physicians, but began as a concerted effort in 2007 through CURE Clubfoot Worldwide and in partnership with CURE’s program in the Dominican Republic. After the devastating earthquake, a multi-institutional effort ensued to rebuild the program involving CURE Clubfoot, Christian Blind Mission (CBM) International, Medical Teams International, A Leg To Stand On (ALTSO), Adventist Hospital, and the Ministry of Health.[18] Most organized clubfoot care in this nation has since been managed by CURE Clubfoot (a program of CURE International, Inc.) in partnership with local institutions and non-governmental organizations (NGOs). With the ongoing expansion of clubfoot care, it is critical to understand the current state of treatment in this low-resource setting to optimize care in Haiti and other similar locations.

We conducted a retrospective cohort study on the 4-year experience at a clubfoot clinic in Port-au-Prince, Haiti. We analyzed patient factors associated with delayed presentation, increased number of casts, and relapse. Finally, we compared treatment patterns and outcomes of the Ponseti method in high and low-resource settings.

## Materials and methods

### Setting

The study site is a free-care CURE Clubfoot clinic established in 2011 in Port-au-Prince, Haiti, at the Hôpital de l’Université d’Etat d’Haiti (HUEH), the largest government hospital in the country. It is the only dedicated clubfoot center in the capital city with a population of nearly 1 million, and in combination with a 2^nd^ clubfoot clinic at the Adventist Hospital in Diquini, Haiti, this site is only 1 of 2 dedicated clubfoot clinics serving the entire metropolitan area and beyond with an estimated population of 2.6 million. At present no compensation for travel or local housing is provided though many in Haiti often have some contacts or relatives in and around the capital city. Treatment itself is free for the patient, and the hospital is reimbursed a previously negotiated rate by CURE Clubfoot for each patient depending on level of service provided. Medical supervision is provided by a Haitian pediatric fellowship-trained orthopaedic surgeon and oversight by a senior member (RBC) of the Pediatric Orthopaedic Society of North America (POSNA) committed to clubfoot care in Haiti. Treatment is provided by orthopaedic residents or attendings trained in the Ponseti method. The clinic has a dedicated assistant, or counselor, for clerical and social work related to maintaining records and assisting families. This counselor explains follow-up and performs phone calls for any missed visits.

### The Ponseti Method

**Fig 1:**
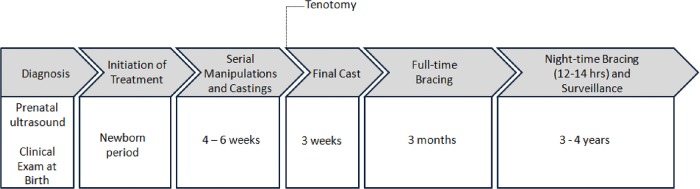
Standard of care for clubfoot diagnosis and treatment in developed nations. Shaded boxes represent sequential phases of care according to the Ponseti Method of non-operative management of clubfoot. White boxes under each phase portray typical time period and duration of each phase.

The Ponseti method has developed over the years into a widespread, minimally invasive protocol for the initial management of clubfoot deformity through its sequential phases of diagnosis (case identification and referral), casting (achieving correction) with possible percutaneous Achilles tenotomy, and bracing (maintaining correction) (Fig 1). In general, treatment is recommended to begin within the first month of life.[19–22] Many groups though have reported varying degrees of success using this method in older patients when necessary. [10, 13, 14, 23–32] After 4–6 weeks of manipulations and weekly castings, the cavus, adduction, and varus deformities are typically corrected, as well as some or all of the equinus deformity.[19, 20, 33–36] Any residual equinus is treated with a percutaneous Achilles tenotomy followed by 3 weeks in a cast.[37] Care providers are trained that complete correction of cavus, adductus, varus should be obtained with only equinus remaining prior to tenotomy, with less than 15 degrees of dorsiflexion as the equinus parameter for tenotomy. To maintain the correction, a foot abduction brace is used full-time immediately after casting for 3 months, followed by night-time bracing for 3–4 years.[19, 20, 22, 36, 38]

During the study period at HUEH, foot correction was maintained with Steenbeek braces (obtained from a local Haitian manufacturer in partnership with Mobility Outreach International (MOI) and BRAC) as well as donated MD Ortho Ponseti Splints.

### Design / Sample

We reviewed medical records of patients presenting between November 2011 and October 2015. Clinic records, modeled after the International Clubfoot Registry, were entered into a REDCap database [39], including baseline demographics (age, sex, place/setting of birth), family history, prior treatment elsewhere, associated anomaly, i.e. physical exam abnormalities (spine, hip, upper extremity, lower extremity, neurologic), diagnosis (idiopathic, syndromic, neuropathic, recurrent, postural, metatarsus adductus [MTA]), laterality, Pirani score (0–6), treatment type (cast, tenotomy, brace), and complications (yes/no). The study dataset is provided as a supplemental file (S1 Table). Analytic outcomes evaluated include delayed presentation, number of casts required, and relapse. To avoid overestimating the number of casts, we excluded visit dates with no accompanying information, presumed to be no-shows. In our clinic, patients are casted for 3 weeks after tenotomy, with this time included as part of the “casting phase” / “duration of casting” in the analysis. Relapse is defined as a return to cast application anytime after initiation of bracing. We utilized deductive imputation to enter missing dates based on the clinic’s operating schedule and visits before/after each missing field.

### Data elements

We dichotomized several continuous variables and created categorical variables based on clinical relevance and frequency distribution. Modeling a prior study in an Indian cohort [17], we defined delayed presentation as initial age ≥ 6 months as treatment started after this age may result in full time bracing extending into walking age, and to ensure adequate numbers in each group for analysis. Hometown was dichotomized into Port-au-Prince or not. For place of birth, we distinguished clinic/hospital vs. home vs. missing. Associated physical abnormalities were dichotomized into yes/no. For patients with bilateral clubfoot, we used the higher Pirani score.

### Comparing to North American standards of care

We compared patient data from HUEH to typical Ponseti treatment patterns reported by a 2012 clubfoot management survey of POSNA members.[40] We compared treatment patterns and outcomes of age at presentation, number of casts, duration of casting, tenotomy rates and relapse rates. We derived the number of casts in the POSNA data by dividing the mean duration of the casting phase with the mean duration of each cast. To include Haitian patients most similar to the scenario queried by the POSNA survey, we used data from idiopathic clubfeet patients who received at least 3 castings and had documented evidence of a brace after casting.

### Statistical analysis

We calculated descriptive statistics as percentages for categorical variables or as means (± standard deviation [SD]) or medians (25^th^ and 75^th^ percentiles) for continuous variables based on distribution. Since odds ratios often overestimate the risk ratios for common outcomes, we utilized a modified Poisson regression approach to estimate the adjusted relative risk (RR) and 95% confidence intervals (CI) for each covariate.[41] We advanced to multivariable analyses those variables that had relative risks of >1.5 or <0.67. We selected the final model by assessing both statistical significance and clinical relevance, focusing on factors available at initial presentation. We found significant colinearity between associated anomaly and diagnosis variables in the model for risk factors for increased number of casts, and for clinical relevance, diagnosis is included in the final model rather than associated anomaly.

When selecting our study sample, we took an approach to evaluate all-comers to the clubfoot clinic and included all diagnoses in the analysis. We adjust for diagnosis in our model with a dichotomous variable of idiopathic clubfoot versus all others, as we felt isolating idiopathic clubfoot to be the most clinically relevant diagnosis, and these results are presented here. However, to further examine any effects of including MTA and postural clubfoot in our sample, after the all-comer analysis, we also repeated the analysis with a diagnosis variable isolating MTA and postural clubfoot versus all other diagnoses.

Analyses were conducted using SAS software.[42] All reported p-values are two-sided, and p-values <0.05 were considered statistically significant.

### Ethics statement

The study was approved by the institutional review board at Partners Healthcare and performed with the consent and approval of both CURE and local HUEH administration. Patient records were anonymized and entered into a REDCap database.

## Results

### Study sample

The study sample was comprised of 168 children (257 feet). Age at presentation ranged from 0 days (birth)– 4.4 years old, with 20% of children presenting at or later than 6 months of age and 8% at or later than 1 year (Table 1). Excluding missing/blank fields, most children were male (62%), 63% had bilateral disease, and 35% had an associated congenital anomaly. Approximately half (46%) presented with idiopathic clubfoot, 23% syndromic, 13% postural, 5% metatarsus adductus, 1% neuropathic, 1% recurrent, and 11% unknown/missing. Overall the mean Pirani score at presentation was 4.6 (± 1.8), median score 6.0 (25^th^ to 75^th^ %ile 3.0–6.0). Most patients resided in Port-au-Prince (83%), over one third of children (35%) were delivered at home, and 16% reported prior (failed) treatment for clubfoot elsewhere.

**Table 1:** Factors at presentation associated with delayed age at presentation (≥ 6 months old).

			Delayed (≥ 6 mo) Age at Presentation				Crude		Adjusted		
	Total		Yes		No		RR	95% CI	RR	95% CI	*p-value*
	N	%	N	%	N	%					
Total no. of patients	168	100	34	20.2	134	79.8	--	--	--	--	--
Sex											
Female	59	35.1	14	23.7	45	76.3	1.00	Ref.			
Male	95	56.6	19	20.0	76	80.0	0.84	0.46–1.55			
Missing	14	8.3	1	7.1	13	92.9	0.30	0.04–2.10			
Laterality											
Unilateral	59	35.1	13	22.0	46	78.0	1.00	Ref.			
Bilateral	99	58.9	17	17.2	82	82.8	0.78	0.41–1.49			
Missing	10	6.0	4	40.0	6	60.0	1.82	0.74–4.46			
Associated abnormality											
No	109	64.9	18	16.5	91	83.5	1.00	Ref.	1.00	Ref.	
Yes	59	35.1	16	27.1	43	72.9	1.64	0.91–2.98	1.62	0.80–3.29	0.18
Idiopathic Diagnosis											
No	90	53.6	19	21.1	71	78.9	1.00	Ref.			
Yes	78	46.4	15	19.2	63	80.8	0.91	0.50–1.67			
Family history of clubfoot											
No	163	97.0	33	20.3	130	79.8	1.00	Ref.			
Yes	5	3.0	1	20.0	4	80.0	--	--			
Patient is first-born child											
No	85	52.5	15	17.7	70	82.4	1.00	Ref.			
Yes	77	47.5	18	23.4	59	76.6	1.32	0.72–2.44			
Prior treatment for clubfoot											
No	103	61.3	12	11.7	91	88.4	1.00	Ref.	1.00	Ref.	
Yes	19	11.3	13	68.4	6	31.6	5.87	3.18–10.84	6.33	3.18–12.62	<0.001
Missing	46	27.4	9	19.6	37	80.4	1.68	0.76–3.71	0.95	0.28–3.20	0.93
Place of birth											
Hospital	100	59.5	19	19.0	81	81.0	1.00	Ref.			
Home	54	32.1	14	25.9	40	74.1	1.36	0.74–2.50			
Missing	14	8.3	1	7.1	13	92.9	0.38	0.05–2.59			
Port au Prince native											
No	24	14.3	6	25.0	18	75.0	1.00	Ref.			
Yes	120	71.4	26	21.7	94	78.3	0.87	0.40–1.88			
Missing	24	14.3	2	8.3	22	91.7	0.33	0.07–1.49			
	**N**	**Mean (SD)**	**N**	**Mean (SD)**	**N**	**Mean (SD)**					
Pirani score at presentation, per 0.5 unit decrease	151	4.6 (1.8)	25	4.2 (1.6)	126	4.7 (1.8)	1.07	0.99–1.15	1.09	1.00–1.19	0.051

N = 168 for bivariate, N = 151 for multivariable analysis. SD = standard deviation, RR = Risk Ratio, CI = confidence interval, IQR = interquartile range, Ref = Reference.

### Factors associated with delayed presentation

On a bivariate level, the only risk factor significantly associated with delayed presentation was having prior treatment elsewhere (RR 5.87, 95% CI 3.18–10.84) (Table 1). Other potentially important risk factors included the presence of an associated anomaly (RR 1.64, 95% CI 0.91–2.98), a lower Pirani score (RR 1.07 per 0.5 decrease in score, 95% CI 0.99–1.15), and home birth versus hospital birth (RR 1.36, 95% CI 0.74–2.50). In multivariable analysis, prior treatment (RR 6.33, 95% CI 3.18–12.62) was significantly associated with delayed presentation. Adjusting specifically for MTA and postural clubfoot patients together versus all other diagnoses did not affect these statistical conclusions.

### Factors associated with increased number of casts

In multivariable analysis, children presenting with a non-idiopathic diagnosis were 2.28 times more likely (95% CI 1.08–4.83) to require ≥10 casts compared to patients with isolated clubfoot. Children born at home were 1.61 times more likely (95% CI 0.65–3.99), and those with an unknown/missing place of birth were 3.38 times more likely (95% CI 1.48–7.76) compared to those born in a hospital setting. Furthermore, with every 0.5 unit increase in Pirani score at presentation, the risk of requiring ≥10 casts more than doubled (RR 2.78, 95% CI 1.17–6.64) (Table 2). Having an associated anomaly is associated with requiring increased number of casts in a univariate analysis (RR 3.22, 95% CI 1.30–8.01) but was not included in the multivariable model due to significant colinearity with diagnosis. At the time of entering a brace, mean Pirani score was 0.63 (SD 0.64), median 0.5 (25^th^ to 75^th^ %ile 0.0–1.0). In a secondary analysis dichotomizing MTA and postural clubfoot patients together versus all others, MTA and postural patients did receive statistically significantly fewer casts.

**Table 2:** Factors associated with 10 or more casts needed to treat clubfoot deformity.

	Number of Casts				Crude		Adjusted		
	≥ 10		< 10		RR	95% CI	RR	95% CI	*p-value*
	N	%	N	%					
< 6 months age at presentation									
No	1	8.3	11	91.7	1.00	Ref.			
Yes	15	19.7	61	80.3	2.37	0.34–16.33			
Male Gender									
No	8	23.5	26	76.5	1.00	Ref.			
Yes	7	14.6	41	85.4	0.62	0.25–1.55			
Bilaterality									
No	5	11.9	37	88.1	1.00	Ref.			
Yes	11	22.9	37	77.1	1.93	0.73–5.09			
Any associated abnormality									
No	6	10.3	52	89.7	1.00	Ref.			
Yes	10	33.3	20	66.7	3.22	1.30–8.01			
Idiopathic Diagnosis									
Yes	7	13.5	45	86.5	1.00	Ref.	1.00	Ref	
No	9	23.7	29	76.3	1.76	0.72–4.30	2.28	1.08–4.83	0.03
Family history of clubfoot									
No	16	20.0	64	80.0	--	--			
Yes	0	0	4	100	--	--			
Patient is the first-born child									
No	7	15.2	39	84.8	1.00	Ref.			
Yes	9	21.4	33	78.6	1.41	0.58–3.45			
Prior treatment for clubfoot									
No	11	19.3	46	80.7	1.00	Ref.			
Yes	3	27.3	8	72.7	1.41	0.47–4.25			
Place of birth									
Hospital	6	11.1	48	88.9	1.00	Ref.	1.00	Ref.	
Home	6	22.2	21	77.8	2.00	0.71–5.62	1.61	0.65–3.99	0.30
Missing	4	44.4	5	55.6	4.00	1.40–11.43	3.38	1.48–7.76	0.004
Port au Prince native									
No	2	20.0	8	80.0	1.00	Ref.			
Yes	12	16.9	59	83.1	0.85	0.22–3.24			
Relapse									
No	4	7.7	48	92.3	1.00	Ref.			
Yes	12	32.4	25	67.6	4.22	1.48–12.05			
	**N**	**Mean (SD)**	**N**	**Mean (SD)**					
Pirani score at presentation, per ↑ 0.5 increment	16	6.0 (0.1)	73	4.4 (1.7)	3.27	1.16–9.19	2.78	1.17–6.64	0.02

Exclusions included those who were not casted (n = 20) and those who were lost to early bracing follow-up (n = 63). SD = standard deviation, RR = Risk Ratio, CI = confidence interval.

### Factors at presentation associated with relapse

The adjusted multivariable analysis demonstrated that even adjusting for severity/Pirani score, male patients to have a 35% lower risk of relapse than females (RR 0.65, 95% CI 0.43–0.99), and children with higher Pirani score at presentation to have a higher risk of relapse (RR 1.09 per 0.5 increase, 95% CI 1.00–1.17) (Table 3). We did not find that associated abnormality, non-idiopathic diagnosis, home birth, or residence outside Port-au-Prince were associated with relapse in this sample. Adjusting specifically for MTA and postural clubfoot patients together versus all other diagnoses did not affect these statistical conclusions.

**Table 3:** Factors at presentation associated with relapse.

	Relapse				Parameter Estimates		Adjusted Estimates		
	Yes		No		RR	95% CI	RR	95% CI	*p-value*
	N	%	N	%					
<6 months at presentation									
No	5	31.3	11	68.8	1.00	Ref.			
Yes	41	45.1	50	55.0	1.44	0.67–3.09			
Male Sex									
No	23	56.1	18	43.9	1.00	Ref.	1.00	Ref.	
Yes	21	37.5	35	62.5	0.67	0.43–1.03	0.65	0.43–0.99	0.04
Missing	3	25.0	9	75.0	0.45	0.16–1.23	0.45	0.16–1.23	0.12
Bilaterality									
No	22	46.8	25	53.2	1.00	Ref.			
Yes	25	40.3	37	59.7	0.86	0.56–1.32			
Any associated abnormality									
No	29	41.4	41	58.6	1.00	Ref.			
Yes	17	46.0	20	54.1	1.11	0.71–1.73			
Idiopathic Diagnosis									
No	21	42.0	29	58.0	1.00	Ref.			
Yes	26	44.1	33	55.9	1.05	0.68–1.62			
Family history of clubfoot									
No	44	44.9	54	55.1	1.00	Ref.			
Yes	2	50.0	2	50.0	1.11	0.41–3.04			
Patient is the first-born child									
No	23	43.4	30	56.6	1.00	Ref.			
Yes	24	44.4	30	55.6	1.02	0.67–1.57			
Prior treatment for clubfoot									
No	31	43.7	40	56.3	1.00	Ref.			
Yes	6	46.2	7	53.9	1.06	0.56–2.01			
Missing	10	40.0	15	60.0	0.92	0.53–1.58			
Place of birth									
Clinic	24	39.3	37	60.7	1.00	Ref.			
Home	17	44.7	21	55.3	1.14	0.71–1.82			
Missing	6	60.0	4	40.0	1.53	0.84–2.76			
Port au Prince native									
No	4	33.3	8	66.7	1.00	Ref.			
Yes	35	42.2	48	57.8	1.27	0.55–2.93			
Missing	8	57.1	6	42.9	1.71	0.68–4.30			
	**N**	**Mean (SD)**	**N**	**Mean (SD)**					
Pirani score at presentation, per ↑ 0.5 increment	47	5.0 (1.5)	61	4.3 (1.8)	1.08	1.00–1.17	1.09	1.00–1.17	0.038

Exclusions included those who received less than one cast or did not reach bracing phase of care (i.e. loss to follow-up) (n = 109). SD = standard deviation, RR = Risk Ratio, CI = confidence interval.

### Standard of care comparisons

**Fig 2:**
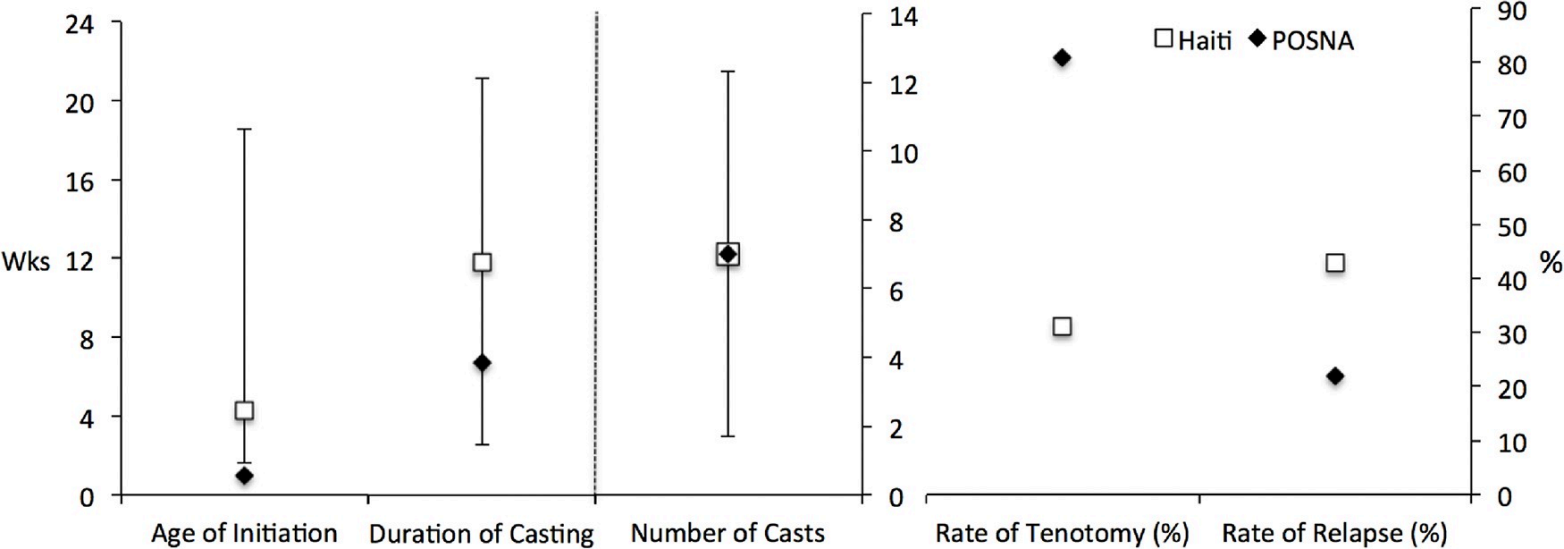
Comparison of North American orthopaedic survey data on clubfoot standard of care with current treatment patterns in Haiti. POSNA = Pediatric Orthopaedic Society of North America. Haiti data presented as median age, mean duration, and mean number of casts. Error bars refer to inter-quartile range (age) and standard deviation (duration and number of casts).

When comparing data from a Haitian clinic with a 2012 POSNA survey on typical clubfoot care parameters, patients in this low-resource setting presented at a later age (median 4.1 [25^th^ to 75^th^ %ile 1.6–18.1] weeks vs. 1 week), had a longer duration of time in the manipulation and casting phase (12.5 [SD 9.8] weeks vs. 7.1 weeks), and had similar average number of casts per person (7 [SD 5.3] vs. 7) (Fig 2). Documented rates of tenotomy appear lower in the developing nation (31%) compared to POSNA respondents (81%), and inversely, relapse rates are higher in this Haitian sample (43%) versus the high-resource setting (22%).

## Discussion

In this report, we analyze four years of experience with the Ponseti method in a low-resource setting, as applied to clubfoot care in Port-au-Prince, Haiti. We provide benchmark metrics and demonstrate gaps in care including delayed presentation, longer duration of casting, and increased relapse rate compared to surveyed standards in high-income countries. Furthermore, in this study sample we identify prior treatment as a risk factor for delayed presentation; non-idiopathic diagnosis, missing/unknown place of birth, and higher Pirani score as risk factors for needing ≥ 10 castings; and female sex and higher Pirani score as risk factors for relapse.

Our finding of alternative/prior treatment as a barrier and risk factor for delayed care is consistent with other reports.[11, 43, 44] While a seemingly intuitive reason for delay, this finding highlights the need for improved screening and referral to experienced treatment centers and providers in a low-resource context. Successful care outside of this referral clinic likely occurs, but the significant and repeated finding of outside treatment as a source of delay warrants further efforts into streamlining care soon after birth towards trained providers with known outcomes. Strategies may include focused efforts to overcome barriers to travel to centralized clubfoot clinics, or consideration of a more de-centralized, even mobile, follow-up model.

The impact of age at presentation on outcomes remains controversial based on conflicting results in the literature.[11, 17, 45, 46] Though traditional teaching has emphasized early initiation of casting [9, 21], recent reports have questioned the importance of immediate care [45, 46] and even recommended waiting until after 1 month of age to facilitate proper casting [47]. While it may not be as critical as once believed to begin casting immediately after birth, it remains in our opinion a logical logistic goal to begin care at least early enough to expect the completion of casting and even full-time bracing prior to a child’s typical age of ambulation both for ease of brace wear and for better compliance. In addition, stigma in developing nations towards children with deformities may warrant earlier intervention so as to complete treatment earlier in life.[16] Particularly in a low-resource setting where the number of casts, the duration of casting, and relapse rates may all be high, clubfoot treatment programs should continue to include early care as an ongoing goal to determine which strategic screening and referral interventions might be implemented.

We found that higher Pirani scores at presentation were associated with increased number of casts as well as relapse, consistent with prior work in India and the United Kingdom.[17, 48] This information can be used when counseling families at presentation to prepare for potentially longer treatment durations, as well as to alert providers and families alike to patients requiring closer surveillance for recurrence after casting. We also note some patients entering into a brace without fully corrected feet based on Pirani scores. Given the clinical suspicion amongst care providers in Haiti of a high rate of arthrogryposis (with an expected increase in cast number and relapse) [49], it is possible that a large subset of non-idiopathic clubfoot patients in our sample had undocumented arthrogryposis. Further inquiry and prospective analysis is warranted into the etiologies of clubfoot in this population, especially given that a non-idiopathic diagnosis is confirmed to require additional care in our sample (higher number of casts). We note in our study sample a very low tenotomy rate despite care providers having been trained in the standard Ponseti Method. The contributing factors to this finding remain unclear in this setting. However, we suspect the relatively lower tenotomy rate in our sample may contribute to the higher relapse rate, with increased training on indications for tenotomy in the Haitian clinics based on these findings and ongoing prospective follow-up to observe for increased tenotomy rate.

Our data suggested that female sex was associated with a higher risk of relapse in our sample. There has not previously been any published correlations between sex and relapse rates, though female sex in Zimbabwe has been reported to be associated with better initial treatment of clubfoot deformity [50]. Our finding warrants further study in this setting to examine potential disparities in care by gender given the absence of any known biologic mechanism to relate sex and relapse.

Our study may have had limited statistical power to detect an association between home birth and poor outcomes, and the presence of an effect, though not statistically significant, may provide preliminary data to justify a larger prospective study analyzing the impact of home birth on clubfoot care in order to determine whether or not this may be an appropriate target of interventions towards improving screening and access to care, including training and improved awareness at the level of midwives and birth attendants. Patients for whom place of birth was missing may include babies who were abandoned, or those from orphanages, in which case there may be other social factors contributing to poor outcomes.

Results of our study should be viewed in light of some limitations, including its retrospective nature. Our results are likely under-reporting the challenges to care in this developing nation as the clinic is situated in a large referral hospital in the capital city and may not be representative of potentially worse outcomes in smaller, unmonitored rural settings. Most of the CURE clubfoot free referral clinics are in cities or larger towns, but given the finding of prior treatment outside of this network for some patients, we acknowledge there must be treatment by varying levels of care providers, possibly in rural as well as more developed areas. Therefore, our sample may have a lower age at presentation and/or select for families with greater access to care.

In addition, our study sample included all-comers as an evaluation of all care provided at this clubfoot referral clinic. This included patients with diagnoses of idiopathic, syndromic, postural clubfoot and metatarsus adductus. Regardless of etiology and diagnosis, patients were typically serially casted and followed and were included in analysis. Given the very small effect size of diagnosis on delayed treatment or relapse, and the quite large effect size on number of casts, we did not feel the inclusion of postural clubfoot and metatarsus adductus would change the statistical conclusions, but it is possible it contributed in part to the overall low tenotomy rate in our sample. We confirmed in a secondary analysis that inclusion of MTA and postural clubfoot patients had no statistical effect on delayed treatment and relapse analyses, and may have caused an underestimation of the strong effect of diagnosis on number of casts, thus only strengthening the finding that idiopathic clubfoot patients required significantly fewer casts than non-idiopathic.

Our study may also be affected by limited follow-up, defined by either failure to reach the bracing phase, or no documented follow-up after entering a brace. Given the risk of recurrence from noncompliance with bracing [51], it is possible our results under-report relapse and its risk factors. This underscores the need for clubfoot programs to emphasize long-term follow-up and address barriers to care. The study clinic already has a system for telephone calls after missed visits, but further support is warranted, particularly in situations where families must travel for extended periods of time. In addition, during the study period, new clinics have emerged in Haiti and may have altered the study population over time by further selecting for families near this urban center over those in rural areas.

We demonstrate here the establishment of a Ponseti clubfoot program in a resource-limited setting. Our results provide support for the creation of dedicated treatment centers to reduce inadequate or failed care elsewhere, ultimately delaying the onset of casting. We verify previously reported risk factors of associated abnormalities and high Pirani score with poorer outcomes, but also identify a potential gender disparity in this population with female patients at greater risk for relapse, warranting further review of the underlying cause of this novel finding. Finally, we advocate that standardized, prospective electronic data entry should be an integral part of any future clubfoot programs in order to allow more accurate quality measures and ongoing research into clubfoot care in the developing world.

## Supporting information

S1 Table Minimal data set. Dataset of final study population used for analysis.
(XLS)
